# Comparative Phylogeography and Phylogeny of Pennah Croakers (Teleostei: Sciaenidae) in Southeast Asian Waters

**DOI:** 10.3390/genes12121926

**Published:** 2021-11-29

**Authors:** Hong-Chiun Lim, Ahasan Habib, Wei-Jen Chen

**Affiliations:** 1 Institute of Oceanography, National Taiwan University, Taipei 10617, Taiwan; hongchiun_01@yahoo.com; 2 Department of Biotechnology, Faculty of Applied Sciences, AIMST University, Sungai Petan 08100, Kedah, Malaysia; 3 Faculty of Fisheries and Food Science, Universiti Malaysia Terengganu, Kuala Nerus 21030, Terengganu, Malaysia; habibuit@gmail.com; 4 Department of Fisheries and Marine Science, Noakhali Science and Technology University, Sonapur 3814, Bangladesh

**Keywords:** phylogeography, phylogeny, population genetics, *Pennahia*, Sciaenidae, Southeast Asian water, *cytochrome b*, Indo-West Pacific

## Abstract

A broad-scale comparative phylogeographic and phylogenetic study of pennah croakers, mainly *Pennahia anea, P. macrocephalus*, and *P. ovata* was conducted to elucidate the mechanisms that may have driven the diversification of marine organisms in Southeast Asian waters. A total of 316 individuals from the three species, and an additional eight and six individuals of *P. argentata* and *P. pawak* were employed in this study. Two genetically divergent lineages each of *P. argentata* and *P. anea* (lineages L1 and L2) were respectively detected from the analyses based on mitochondrial *cytochrome b* gene data. Historical biogeography analysis with a multi-gene dataset revealed that *Pennahia* species most likely originated in the South China Sea and expanded into the eastern Indian Ocean, East China Sea, and northwestern Pacific Ocean through three separate range expansions. The main diversifications of *Pennahia* species occurred during Miocene and Pliocene periods, and the occurrences of lineage divergences within *P. anea* and *P. argentata* were during the Pleistocene, likely as a consequence of cyclical glaciations. The population expansions that occurred after the sea level rise might be the reason for the population homogeneity observed in *P. macrocephalus* and most *P. anea* L2 South China Sea populations. The structure observed between the two populations of *P. ovata*, and the restricted distributions of *P. anea* lineage L1 and *P. ovata* in the eastern Indian Ocean, might have been hampered by the northward flowing ocean current at the Malacca Strait and by the distribution of coral reefs or rocky bottoms. While our results support S. Ekman’s center-of-origin hypothesis taking place in the South China Sea, the Malacca Strait serving as the center of overlap is a supplementary postulation for explaining the present-day high diversity of pennah croakers centered in these waters.

## 1. Introduction

Southeast Asian (SEA) waters encompass the South China Sea, Philippine and Indonesian waters, and extend west to the Andaman Sea and south to the Timor Sea [1]. Although this region represents only about 2.5 per cent of the surface of the world’s ocean, it has the highest species diversity in the world and the divergence among its different ecosystems is high [1,2,3]. Interactions within and between each of its ecosystems are complex and have not been well documented. These features not only have significant impacts on the biogeochemical cycle of the ocean, but are also important considerations in other research such as the biogeography and evolutionary biology of marine organisms. By focusing on benthic and pelagic biotas of coastal and shelf waters and based on literature reviews, Spalding et al. (2007) defined the marine biogeographic regions of the world and attributed 3 realms, 27 provinces, and 77 ecoregions to the Indo-West Pacific (IWP) [4]. It turns out that SEA waters comprise a high number of defined biogeographic units, with 7 provinces and 22 ecoregions. Nevertheless, understanding the processes that have driven the diversification and maintenance of high species diversity in the IWP is crucial for both marine biological research and conservation, yet it is awaiting clarification [5,6].

Several hypotheses have been proposed to explain the extreme concentration of biodiversity of marine species in the Coral Triangle that covers part of SEA waters, among which three are primarily mentioned [7,8]. The ‘Center of Origin’ hypothesis proposes that species originated in the central IWP (SEA waters/Coral Triangle) and expanded their geographic range outwards during different geological time periods [9]. An alternative hypothesis is the ‘Center of Accumulation’ hypothesis, which suggests that the species accumulated in the central IWP were from peripheral regions where speciation mainly occurred [10,11]. Finally, the ‘Center of Overlap’ hypothesis suggests that the central IWP serves as an overlapping area that harbors different species or genetic lineages from adjacent biogeographic regions [12]. Many possible mechanisms at play have been proposed and discussed to support the suggested hypotheses. These mechanisms include the complex geologic history of the Late Cenozoic such as tectonic movement, paleoclimatic and palaeoceanographic events, and the formation of physical or soft barriers [7,12,13,14]. Other than the complex geologic history, the habitat preferences, adult or larval dispersal abilities, mating behaviors, and other factors such as physical oceanography may also have impacted species diversity as well as species distribution patterns in this region [15]. Nevertheless, most studies invoked sea level change over the last few million years resulting in the isolation of small marine basins, changes in currents, new land bridge formations, and changing levels of population connectivity as the common mechanisms that created isolated populations of marine species and subsequently promoted genetic diversification or speciation [8,16,17,18,19,20,21].

To better understand the underlying processes of genetic diversification and speciation, broad-scale biogeographic and phylogenetic surveys of diverse species are thus needed [6,14]. The comparative phylogeographic study that we focus on here provides an important tool in discovering and evaluating the shared historical factors that have driven species evolution and shaped the distribution of biodiversity [22]. That is, when similar phylogeographical patterns are observed in multiple co-distributed species of the same genus or family, the shared historical factors that might be responsible for this similarity can be deduced. In contrast, the discovery of discrepancies may be attributed to several factors such as the effect of physical oceanography [23,24], species specific habitat requirements [25,26], and larval dispersal abilities [27,28,29].

To our knowledge, comparative phylogeographic studies incorporating thorough taxon sampling from SEA waters are essentially unavailable. Carpenter and his colleagues tested the phylogeographic patterns of marine taxa across the Coral Triangle, but their study was based on literature reviews and on distantly related taxa [8]. Previous marine phylogeographic and/or population genetic studies using empirical data from this region actually focused mainly on surveys from individual countries and often with a single species; many of them addressed specific questions related to fisheries management [26,30,31,32,33,34,35]. In the present study, a comparative phylogeographic study concomitant with a thorough phylogenetic survey was conducted on pennah croakers to elucidate the mechanisms underlying the patterns of species/genetic diversification of marine organisms in SEA waters. Pennah croakers (Perciformes, Sciaenidae) are medium-sized demersal fishes inhabiting sandy or muddy bottoms in coastal inlets to a depth of 140 m [36,37]. They range from waters off Tohoku, Japan, to the East China Sea, Yellow Sea, South China Sea, and East Indian Ocean [37,38,39,40]. Currently, five Pennah croaker species, *Pennahia anea* [41], *P. argentata* [42], *P. macrocephalus* [43], *P. ovata* [44] and *P. pawak* [45], are recognized [46]. These fishes contribute a major component of the coastal ichthyofauna, as well as being important constituents of subsistence fisheries in SEA countries [47,48]. Here, we first explored the phylogeny and genetic diversity of *Pennahia* species based on the analysis of DNA sequence variations of a mitochondrial *cytochrome b* (*cytb*) gene. We then reconstructed a time-calibrated phylogenetic tree and inferred the historical biogeography of *Pennahia* species/lineages based on a multi-gene dataset. To complete the comparative phylogeographic and population genetic surveys, three *Pennahia* species, *P. anea*, *P. macrocephalus*, and *P. ovata*, were employed. *Pennahia anea* is distributed across the IWP from the Indian Ocean to the South China Sea, east to the Philippines, and south to Sabah, Sarawak, and Indonesia. Investigation of the widespread *P. anea* across the two oceans may provide insight into the influence of the Indo-Pacific or Sunda Shelf barrier [20] to population differentiation or species diversification of marine species in SEA waters. Another species, *Pennahia ovata*, occurring only in the eastern Indian Ocean, shares a partially overlapping distribution with *P. anea* in the Malacca Strait. This overlapping distribution may provide an opportunity to investigate the common mechanisms such as physical or local oceanographic processes that may act in population connection or differentiation in the two species. Finally, the co-distribution of *Pennahia macrocephalus* and *P. anea* in the South China Sea enables us to conduct a comparative study as well as discover any other plausible physical barriers or faunal boundaries in the South China Sea [4,8] that remain unclear to date.

## 2. Materials and Methods

### 2.1. Ethical Statement

The animal study was reviewed and approved by National Taiwan University in accordance with the National Taiwan University’s guidelines regarding animal research. As this project did not involve experiments on live fishes, no ethics statement is required.

### 2.2. Specimen Collection

**Figure 1:**
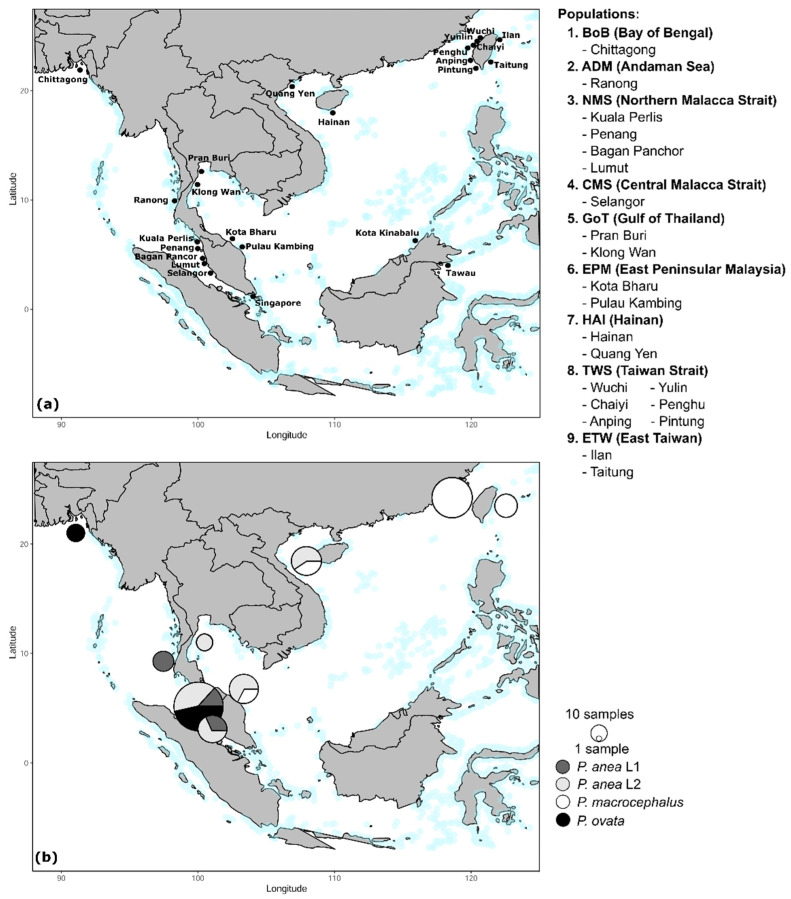
Sampling localities (black dots) and assigned populations (**a**); Species distributions of *P. anea*, *P. ovata*, and *P. macrocephalus* in SEA waters (**b**). Circle size is proportional to the number of samples. Light blue spots represent coral reefs.

Our sampling area extended from the northeastern part of the South China Sea including Taiwan to the Bay of Bengal in the eastern Indian Ocean (Figure 1). Most of the sampling sites were along the coast of Peninsular Malaysia, as it is the boundary between the Indian and Pacific Oceans. This sampling strategy enabled us to test the influence of the Sunda Shelf barrier on the evolution of the studied species. We collected 152 individuals of *P. anea* from both ocean regions and 53 individuals of *P. ovata* from the Bay of Bengal and Malacca Strait. A total of 111 individuals of *P. macrocephalus* were collected from the South China Sea. It should be noted that although the species distribution range covers the Indian Ocean, no individuals of *P. macrocephalus* were found from the collection sites in the eastern Indian Ocean despite several sampling attempts. In total, 316 individuals of *Pennahia* species were collected from 24 sites (Figure 1). Samples from adjacent locations were pooled and assigned into a single population; samples from Kota Kinabalu and Tawau, Sabah, and Singapore were omitted from population level analyses due to the number of samples per site being too low. In total, nine populations were assigned for further comparative phylogeographic and population genetic analyses (Figure 1, sampling details in Supplementary Tables S1 and S2). Specimen identification followed Fishes of Taiwan [49], Food and Agriculture Organization of the United Nations (FAO), and other species identification guides [36,50]. A small piece of muscle tissue or fin clip from each specimen was excised and preserved in 95% ethanol for molecular examination. Freshly collected specimens were photographed prior being fixed in formalin. Most of the voucher specimens were deposited in the National Taiwan University Museums (NTUM), Taipei, and Centre for Marine and Coastal Studies (CEMACS), Universiti Sains Malaysia, Penang (Table S2). 

### 2.3. DNA Extraction, PCR Amplification, and Sequencing

**Figure 2:**
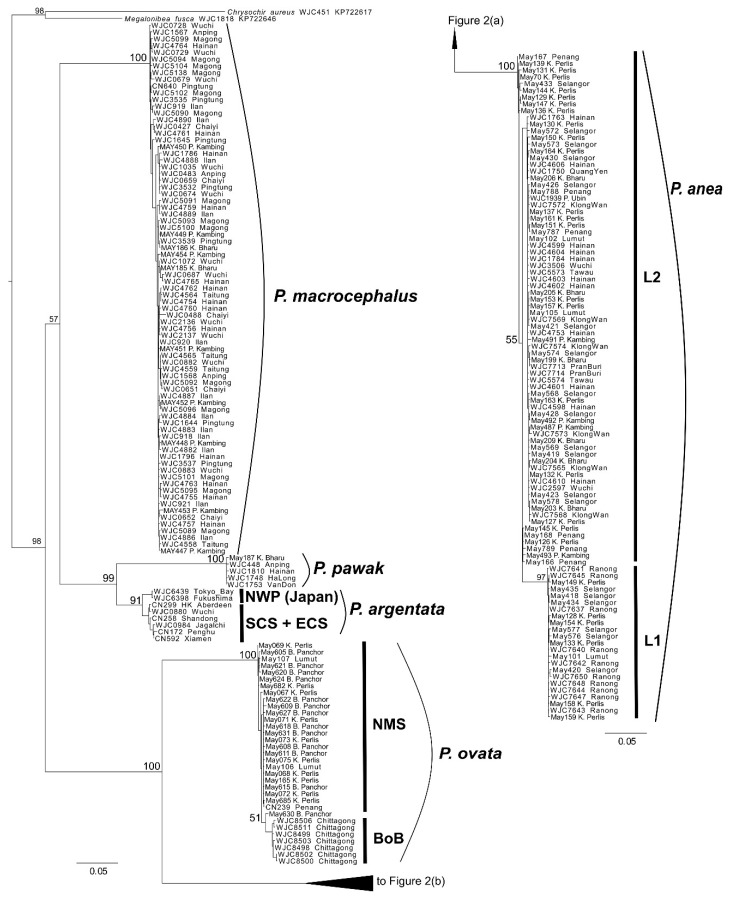
(**a**) *Pennahia* maximum likelihood phylogenetic tree based on *cytb* gene. Branch length is proportional to the inferred number of nucleotide substitutions. Numbers at nodes represent bootstrap values in percentages. Values < 50% are not shown. Tree was rooted using *Chrysochir aureus* and *Megalonibea fusca*; (**b**) Continuous of Figure 2a.

Total genomic DNA was extracted from muscle tissue using an automated LabTurbo 48 Compact System extractor (Taigene Biosciences Corp., Taipei, Taiwan) and LGD 480-220 DNA-extraction kits (Taigene Biosciences Corp., Taipei, Taiwan) following manufacturer protocols. Polymerase chain reactions (PCR) were conducted to amplify the partial mitochondrial DNA (mtDNA) *cytochrome b* (*cytb*) gene for all samples. The *cytb* gene was chosen as it contains both slowly and rapidly evolving codon positions, which are useful in producing robust phylogenies at species and genus levels [32,51]. In addition, one to five representative samples from each species and lineage based on the reconstructed *cytb* gene tree (Figure 2) and the biogeographic regions were further PCR amplified for a mitochondrial *cytochrome oxidase subunit I* (*COI*) gene and exon 3 of a nuclear *recombination activating gene 1* (*RAG1*) for time tree reconstruction (see details in the later section). These gene markers were selected for their ability to provide valuable phylogenetic information based on previous studies investigating Sciaenidae systematics [48,52] and other marine fishes [53,54,55]. Details of the representative samples and the primers used are provided in Tables S3 and S4. Each PCR reaction contained 1–2 µL DNA template (5–30 ng), 1X EmeraldAmp MAX HS PCR Master Mix (Takara Bio USA, Inc., San Jose, CA, USA), 0.1 µL of each primer, and sterile distilled water added to make a final reaction volume of 12.5 µL. The thermal profile of the PCR was as follows: initial denaturation at 95 °C for 4 min, followed by 35 cycles of denaturation at 95 °C for 40 s, annealing temperature at 56 °C for *cytb* and *RAG1* and 51 °C for *COI* for 40 s, elongation at 72 °C for 90 s and a final elongation at 72 °C for 5 min. PCR products were then checked on 1% agarose gel for the presence of successfully amplified PCR products, and then purified using the AMPure magnetic bead clean-up protocol (Agencourt Bioscience Corp., Beverly, MA, USA). PCR products were sent for sequencing at Genomics BioScience and Technology Co., Taipei, Taiwan, using an ABI 3730 analyzer (Applied Biosystems, Foster City, CA, USA). For longer amplicons (*cytb* and *RAG1*), gene fragments were sequenced bidirectionally in order to assemble robust sequences. GenBank accession numbers for the sequences used in this study appear in Supplementary Table S2.

### 2.4. Sequence Alignment and Datasets 

The resulting DNA sequences were assembled and edited using CodonCode Aligner v.8.0.2 (CodonCode Corporation, Dedham, MA, USA). Sequences (usually at both ends) with low quality or problematic base calls (below Q [phred quality value] 20) were verified by eye and trimmed. For *RAG1* gene sequences, observed heterozygous sites were coded following the International Union of Pure and Applied Chemistry (IUPAC) nucleotide code. Edited sequences were aligned and trimmed to a common length in MEGA7 using the MUSCLE algorithm [56]. Alignment was subsequently checked by eye and adjusted manually when necessary.

The datasets compiled and used for molecular analyses included (1) a complete *cytb* dataset comprising the *cytb* sequences from all collected 316 samples, plus 8 and 6 samples of *P. argentata* and *P. pawak*, respectively, obtained from Lo et al. [48,52]; (2) individual datasets for the *cyt b* sequences of *P. anea*, *P. ovata*, and *P. macrocephalus*; and (3) a combined (or multi-gene) dataset (*cytb*, *COI*, and *RAG1*) comprising the sequences from representative samples of each species/lineage based on the reconstructed *cytb* gene tree and biogeographic regions.

### 2.5. Phylogenetic Inference, Haplotype Networks, and Genetic Distance

To ensure that only the individuals of putative species were selected and to discover the possible existence of cryptic lineages, phylogenetic inference was first conducted on the complete *cytb* dataset. To further test the phylogenetic hypothesis resulted from the inferred *cytb* tree and suggested by previous studies [48,52], the advanced analysis was conducted on the combined gene dataset. The partitioned maximum-likelihood (ML) method implemented in the sequential and parallel program RAxML Version 8 [57] was used for phylogenetic tree reconstruction. The partition was set by gene and codon position. The nucleotide substitution model GTR+G was employed for the analyses because RAxML only provides GTR-related models of rate heterogeneity. To root the reconstructed tree, homologous sequences of two sciaenid species, *Chrysochir aureus* and *Megalonibea fusca*, were chosen as outgroups. Five independent runs were conducted and the final tree with the best ML score was selected among the five ML trees of these runs. Nodal support was assessed with bootstrapping (BS) under the ML criterion based on 1000 pseudo-replicates generated from each of five separate runs. All RAxML analyses with bootstrapping were conducted with the CIPRES Science Gateway at http://www.phylo.org. Phylogenetic trees were visualized and edited using FigTree v1.4.0 [58]. In addition, the mean genetic *p*-distances of *cytb* genes among the populations, species, and lineages detected in our phylogenetic results were calculated using MEGA7 [56].

To detect the phylogeographic structure and visualize the relatedness among haplotypes for each species, haplotype networks based on individual *cytb* datasets were constructed with the median-joining method using PopART (University of Otago, http://popart.otago.ac.nz). Prior to this, haplotypes of each species were first determined using DnaSP v.5.10.1 [59].

### 2.6. Genetic Diversity, Population Differentiation, and Demographic Analyses

Arlequin v. 3.5 [60] was used to estimate genetic diversity, including nucleotide diversity (*π*) and haplotype diversity (*h*), as well as population differentiation and demography on *cytb* datasets. The extent of genetic differentiation between populations within each species/linage was estimated using Φ_ST_ with *p*-distance measurements. Significance was tested by permutation (*N* = 100,000), and *p*-values adjusted with the False Discovery Rate (FDR) procedure [61]. Hierarchical analyses of molecular variance (AMOVA) [62] were conducted to estimate variance components for individuals within populations, among populations within groups, and among groups (Φ_CT_). The grouping scheme (e.g., eastern Indian Ocean group vs South China Sea group) that was best aligned with the genetic structure of a species will result in the greatest amount of variance in the data explained among groups (Φ_CT_). The demographic history of populations was assessed by statistical measurements, deviation from the neutral expectation was tested through Tajima’s D [63] and Fu’s Fs [64] estimations using Arlequin v. 3.5, and significance was tested with 100,000 permutations.

### 2.7. Divergence Time Estimation

Divergence times for *Pennahia* species were estimated to provide a time frame for revealing their evolutionary history based on a Bayesian approach, which incorporated a relaxed molecular clock method and calibration points as implemented in BEAST 2 [65]. The combined gene dataset was used for divergence time estimation. *Chrysochir aureus* and *Megalonibea fusca* were chosen as outgroups. Analyses in BEAST 2 were run three times for 10,000,000 Bayesian MCMC generations each to ensure independent convergence. We used the same GTR + G model of sequence evolution for each partition in BEAST 2 analysis as it was in the RAxML analysis. Substitution rate parameters, rate heterogeneity model, and base frequencies were unlinked across partitions. Previously, Lo et al. (2015) [48] established a robust time-calibrated phylogenetic tree of the Sciaenidae with one sparid and eight sciaenid fossils. We used two of the inferred time points to calibrate our time tree. Here, a single prior age constraint for the root of the tree was set at 13.6 million years ago (Mya) (95% confidence intervals: 10.3–16.9 Mya). An internal calibration point within the *Pennahia* was also set to fix the split time of *P. argentata* and *P. pawak* at 5.5 Mya (95% confidence intervals: 2.3–8.9 Ma) by following a normal distribution. 

Trees and other estimates were sampled once every 100 generations and the parameters of each run were checked for convergence with Tracer v. 1.6 [66]. If the effective sample size (ESS) reached >200 for all parameters [67], it indicates MCMC convergence has been achieved. We removed the burn-in portion (=20% of saved trees) of each run and combined the remaining trees from each run using LogCombiner v2.1.2 [65]. We reconstructed the maximum clade credibility tree with mean divergence times, using TreeAnnotator v. 2.1.2 [65]. The reconstructed time-calibrated tree was visualized and edited using FigTree v1.4.0 [58].

### 2.8. Ancestral Area Reconstruction

The origin and patterns of geographical diversification of *Pennahia* species were assessed through ancestral area reconstruction based on the Dispersal-Extinction-Cladogenesis model (DEC) using Lagrange [68,69]. Four biogeographical regions were defined based on the land constraint, ocean basins and genetic diversification reported from present and previous studies [52,70]. They are the eastern Indian Ocean (EIO), South China Sea (SCS), East China Sea (ECS), and northwestern Pacific off Japan (NWP/Japan). When the geographic range of a species encompassed two or more of these biogeographical ensembles, at least one sample from each was selected so that the entire range of the species was represented in the analysis (Table S3). Default options were selected for other parameters.

## 3. Results

### 3.1. Phylogeny

We analyzed 330 *cytb* sequences, 22 *COI* sequences, and 23 *RAG1* sequences from our collected *Pennahia* samples, which included published sequences from our previous study [48,52]. Characteristics of the individual gene datasets are presented in Table 1. No stop codons or nuclear mitochondrial pseudogenes were detected after the protein coding regions were translated into amino acids as a check. 

Figure 2 shows the reconstructed maximum likelihood (ML) tree based on the complete *cytb* dataset. In this tree, all five *Pennahia* species were confirmed to be monophyletic. Most of their inter-relationships were fully resolved (BS = 98–100%) except the sister-group pair, *P. macrocephalus* with *P. pawak* and *P. argentata* (BS = 57%). At the intraspecific level, no obvious genetic splits were observed within *P. macrocephalus* and *P. pawak*. However, differentiated genetic groups were detected in three other species, notably *P. anea*. Lineage 1 (L1) of *P. anea* consisted of samples collected exclusively from the eastern Indian Ocean (populations ADM, NMS, and CMS; Figure 1 and Figure 2). Lineage 2 (L2) of *P. anea* consisted of samples collected from both the EIO (except the ADM population) and SCS. For *P. argentata*, the two genetic groups consisted of samples collected from the SCS and ECS, and the samples collected from Japan in the NWP/Japan. For *P. ovata*, a nested clade containing the samples from the Bay of Bengal was detected (Figure 2). Based on the mean genetic *p*-distance of the *cytb* gene (Table S5), the divergence between the two groups in *P. anea* and in *P. argentata* were around 4% and 2.2%, respectively. The mean genetic distance between the samples of *P. ovata* from the Bay of Bengal and the NMS population was 1.7%. Nevertheless, these divergences were still much less than the mean divergence between species, which ranged from 11% to 21%. As these divergences might not represent the distinct species, we provisionally consider the separated genetic groups as different cryptic lineages within species (e.g., L1 and L2 in *P. anea*).

### 3.2. Haplotype Network

**Figure 3:**
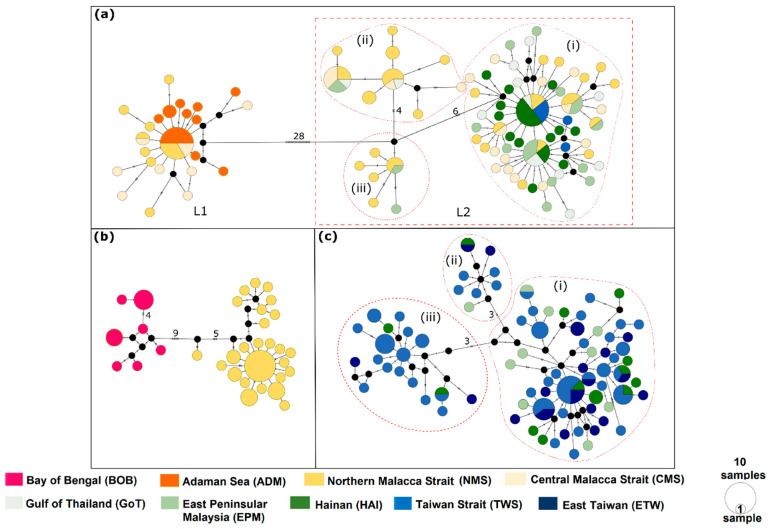
Median-joining haplotype networks of *P. anea* (**a**), *P. ovata,* (**b**) and *P. macrocephalus* (**c**) at the *cytb* gene. The number of mutations is indicated by crossbars and numbers on connecting lines. Circle sizes are proportional to haplotype frequency. Small black dots are missing haplotypes linking the haplotype. Color tones differ according to the nine populations considered in this paper (see Table S1).

Figure 3 illustrates the reconstructed haplotype networks based on individual *cytb* datasets. The numbers of haplotypes detected for *P. anea*, *P. ovata*, and *P. macrocephalus* were 96, 33, and 79, respectively. Network results were concordant with inferred phylogeny, in which the haplotypes of *P. anea* and *P. ovata* can be divided into two major haplogroups within the species where most of the haplotypes were population- or region-specific. For *P. anea*, the L1 haplogroup that consisted of the haplotypes from ADM, NMS, and CMS populations (all located in the eastern Indian Ocean) was separated by 28 mutation steps from the L2 haplogroup consisting of haplotypes from EIO and SCS populations (Figure 3a). Further inspection within the L2 haplogroup revealed three internal haplogroups (L2; i, ii, and iii), which were separated from each other by four to ten mutation steps. For *P. ovata*, two haplogroups corresponding to BoB and NMS populations were separated by nine mutation steps (Figure 3b). One major haplogroup was detected in *P. macrocephalus* (Figure 3c). Similar to L2 of *P. anea*, three internal haplogroups (separated by three to seven mutation steps) without a clear geographic association were observed when a closer inspection was made.

The phylogenetic inference and results from the haplotype networks revealed a co-existence of two genetically differentiated lineages of *P. anea* (L1 and L2) in the NMS and CMS populations at the Malacca Strait. Therefore, the individuals in these populations were treated as samples from two operational taxonomic units (OTUs), herein defined as NMS L1, CMS L1, NMS L2, and CMS L2, with respect to the two OTUs for further phylogeographic and population genetic analyses.

### 3.3. Genetic Diversity, Population Demography, and Population Structure

The haplotype diversity (*h*) of the *cytb* gene ranged from 0.8667 to 0.9857 in *P. anea* populations, 0.8636 to 0.9354 in *P. ovata* populations, and 0.9856 to 1.0 in *P. macrocephalus* populations, while nucleotide diversity (*π*) ranged from 0.0020 to 0.0085 in *P. anea*, 0.0044 to 0.0047 in *P. ovata*, and 0.0068 to 0.0088 in *P. macrocephalus* (Table 2). In general, most populations presented high haplotype diversity and low nucleotide diversity, which was a signature of sudden population expansion after a recent bottleneck event [71]. The population expansion event was further supported by neutrality tests where negative Tajima’s D values were observed in all populations (Table 2). Although only five of the Tajima’s D values were significant statistically, Fu’s Fs, which is more sensitive in comparison to Tajima’s D in detecting demographic expansion in small populations, showed significant negative values in all tested populations except in one population each of *P. macrocephalus* and *P. ovata*.

Table 3 shows the measures of genetic differentiation between populations of each examined species/lineage. No significant population structure was detected among populations within *P. anea* L1 (ADM, NMS L1, and CMS L1; Table 3a). However, within *P. anea* L2, the Hainan (HAI) population was significantly differentiated from others in all pairwise comparisons with Φ_ST_ value ranging from 0.0667 to 0.2765 (Table 3b). The NMS L2 population also showed a high degree of population differentiation to the others, with Φ_ST_ value ranging from 0.0443 to 0.2765; however, only two of the pairwise comparisons were significant. For *P. ovata*, the comparison between BoB and NMS populations revealed a high and significant population differentiation with a Φ_ST_ value of 0.5000 (Table 3c). No population differentiation was found in *P. macrocephalus*, where all pairwise comparisons showed low and non-significant Φ_ST_ values in pairwise comparisons (Table 3d).

The results from AMOVA analyses (Table 4) once again confirmed the presence of two different lineages in *P. anea*. A high and significant Φ_CT_ value of 0.8404 was obtained when groupings based on delineated lineages (L1 and L2) were assigned. Further AMOVA analyses (grouping by ocean regions, ecoregions, and genetic groups) were conducted. These analyses aimed to test the influence of the Sunda Shelf barrier to population differentiation or detect phylogeographic breaks of *Pennahia* species in SEA waters. A non-significant Φ_CT_ value of 0.1224 for the grouping associated with the putative effect of the Sunda Shelf barrier (EIO and SCS groups) was observed in *P. anea* L2. Further separation of the HAI population of *P. anea* L2 into its own group also revealed a low and non-significant Φ_CT_ value of 0.070. For *P. macrocephalus*, although the Φ_ST_ values are low and non-significant, two different groupings were still assigned to test the delimitation of the defined ecoregions by Spalding et al. (2007) [4] and the genetic groups within the South China Sea inferred from our study. The analyses of all hypothesized groupings returned low and non-significant Φ_CT_ values (Table 4).

### 3.4. Reconstruction of Historical Biogeography

**Figure 4:**
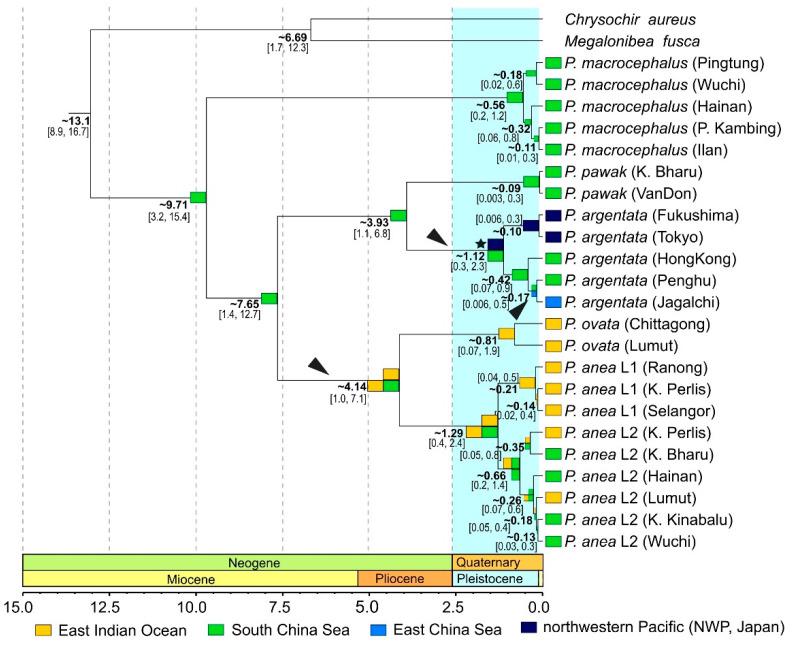
Most-likely ancestral range reconstruction of *Pennahia* species using the Dispersal-Extinction-Cladogenesis (DEC) model onto the simplified Bayesian phylogenetic chronogram constructed in this study. Horizontal timescale is in millions of years (Mya). Numbers at nodes represent time to the Most Recent Common Ancestor (tMRCA) and the 95%HPD is in braces. Most-likely ancestral range reconstruction is indicated by color boxes next to nodes. The light-blue vertical bar indicates the duration of the Pleistocene. Black arrowheads indicate inferred events of range expansions. Black star indicates subsequent allopatric cladogenesis.

In the reconstructed time tree, *P. macrocephalus* was placed as the basal clade of Pennah croakers, followed by two reciprocal clades comprising two sister-group pairs, *P. pawak/P. argentata* and *P. ovata/P. anea* (Figure 4). Although the topology of the multi-locus time tree is different from the *cytb* tree (Figure 2), it is congruent with the findings from our previous study using a multi-gene dataset [52] and the presently reconstructed multi-gene tree based on both ML and Bayesian methods (Figure S1). Thus, we used the present time tree for the further biogeographic analyses as the multi-gene tree should be more robust compared to the single gene tree. The results from the divergence time estimation and ancestral area reconstruction indicated that *Pennahia* species originated and diversified in the SCS during the Middle Miocene to Early Pliocene before undergoing two major range expansions into the EIO and NWP/Japan (Figure 4). The third range expansion occurred during the late Pleistocene into the ECS. Intraspecific diversifications occurred independently during the Middle Pleistocene around 1.2 Mya in *P. anea* and *P. argentata*.

## 4. Discussion

### 4.1. Phylogeny, Diversity and Historical Biogeography 

Our phylogenetic inferences included a complete taxonomic sampling of *Pennahia* species from multiple localities across their distribution range. From the *cytb* and multi-gene analyses, we confirm the monophyly of the five recognized *Pennahia* species. Our phylogenetic results reveal also the occurrence of two phenotypically similar but genetically diverged lineages (L1 and L2) each in *P. anea* and *P. argentata*. While the discovery of cryptic lineages in *P. anea* is new, the presence of two genetically distinct lineages in *P. argentata* has been reported by Han et al. (2008) [70]. In their inference, these two lineages, “Chinese” and “Japanese” clades, diverged by 2% and 3% at the *cytb* and control region loci, respectively. Their results are consistent with ours. In *P. ovata*, we also postulate the possible occurrence of cryptic lineages in *P. ovata*, as the BoB population formed a nested clade within the NMS population with moderate support in the inferred *cytb* tree. When referring to the mean genetic divergence of *cytb*, 1.7% of the divergence between the two haplogroups is not very significant compared to the 4% and 2.2% divergences observed in *P. anea* and *P. argentata*, respectively. Nevertheless, more samples of *P. ovata* from different localities in the eastern Indian Ocean are still needed as our investigation involved only two populations with limited samples.

In addition to phylogenetic implications, the present study also provides the first insight into the origin and species diversification of *Pennahia* species. Our results from multi-gene analysis show that the most recent common ancestor of *Pennahia* species most likely inhabited the SCS during the Miocene. The main diversifications occurred during the Miocene and Pliocene periods between ca. 9.71 Mya and 4 Mya. This finding is concordant with several reports where most of the origin and diversification timing of congeneric marine taxa were during Miocene and/or Pliocene periods [8,14,15,18,21,72,73,74]. As the speciation events pre-dated the Pleistocene, sea level fluctuations due to repeated glaciations during the Pleistocene are thus probably not the main driver for species diversification at this level [8,75,76]. Renema et al. (2008) [77] postulated that climatic or oceanographic events during the Miocene and Pliocene may have altered connectivity between populations, and thus may have led to further species range expansions and subsequent speciations. Actually, the common ancestor of *P. anea* and *P. ovata* first expanded its distribution range from the SCS to the EIO between the Late Miocene and Early Pliocene, and splitting of the two species occurred at 4.14 Mya (Figure 4). The gradual tectonic collision of the Australian and Eurasian Plates starting from the same period may also have initiated the speciation process and confined the species’ distributions [18]. The final formation of the Indo-Pacific biogeographic barrier due to the emergence of Sundaland around 2.5 Mya should have further impacted the diversification of marine taxa inhabiting the area [6,8,15]. The exposure of the barrier should have restricted the gene flow among the *P. anea* ancestral populations, and eventually led to the divergence of different genetic lineages and populations within the species. 

### 4.2. Distribution Pattern

The present study updates knowledge on the distribution of the *Pennahia* species, especially the three occurring in SEA waters (Figure 1b). To further inspect the potential bias for the species/lineage distribution due to sampling gaps (i.e., where some localities were not surveyed in this study), we retrieved the distribution information for those *Pennahia* sequences deposited in GenBank and BOLD Systems. Eventually, we managed to retrieve additional information from eight *COI* sequence records in both databases. Among these records, two sequences were from Bangladesh (MN45837; MT012635), one was from Myanmar (MH235684), and one was from Pakistan (MN512018). These four sequences are almost identical to our *P. anea* L1 *COI* sequences. The other four sequences retrieved from the databases are almost identical to our *P. macrocephalus COI* sequences. They were all from West Java, Indonesia (BOLD Systems: FOAH796-08; FOAH797-08; FOAH856-08; FOAM289-10).

Based on our investigations, *Pennahia* lineages except *P. anea* L1 and *P. ovata* were found in the South China Sea. According to the results of our historical biogeographic inference, Ekman’s center-of-origin hypothesis is supported to explain the present-day distribution of the species. That is, the SCS is the origin and diversification centre for *Pennahia* species, and the presence of *Pennahia* species in other regions occurred in three range expansions from its ancestral area toward the EIO, NWP/Japan, and ECS (Figure 4).

Widespread species across the Indian and Pacific Oceans are common in tropical marine fish species [78,79]. Among the *Pennahia* lineages, *P. anea* L2 is the only one found both in the South China Sea and Indian Ocean (the Malacca Strait). Its sibling lineage, L1, is restricted to the Indian Ocean from the Malacca Strait to the Andaman Sea (Figure 1b), and to the Bay of Bengal (Bangladesh) and Arabian Sea (Pakistan) (see above). *Pennahia ovata* is also restricted to the Indian Ocean. The three lineages co-occurring in the Malacca Strait (Figure 1b) is consistent with the ‘Centre of Overlap’ hypothesis because it served as an overlapping zone harbouring high numbers and mixed evolutionary lineages from different biogeographic provinces [12]. Here, we suggest that the Malacca Strait may be a potential secondary contact zone for different marine species or lineages whose ancestral populations might be allopatrically distributed in the Indian Ocean and the South China Sea during glacial periods.

### 4.3. Population Connectivity

Our phylogeographic and population genetic surveys at *cytb* gene revealed a genetic homogeneity in *P. macrocephalus* and most *P. anea* L2 South China Sea populations (Table 3 and Table 4). The same finding was also reported in the other marine fishes *Thamnaconus hypargyreus* [80]; *Thunnus tonggol* [81]; *Pampus chinensis* [82]; *Decapterus maruadsi* [83]. Population expansion that may facilitate wide distribution and present genetic exchange among the populations may explain this. However, the structuring of the HAI population of *P. anea* L2 in all pairwise comparisons has raised our attention. Two population genetic studies of benthopelagic fishes, the Chinese pomfret, *Pampus chinensis* [82] and silver pomfret, *P. argenteus* [84] in the South China Sea have also been discovered to have population structuring, particularly in the Hainan region. The authors reported that the structuring might be due to geographical segregation (separated by the narrow Qiongzhou Strait) and influenced by the complex system of local surface currents. Recently, clear genetic structuring of the Japanese scad, *Decapterus maruadsi*, from a nearby region in northern Vietnam was also reported despite the genetic homogeneity across the populations in the central IWP [85]. Here, we postulate that the structuring of the HAI population may have resulted from short-distance dispersal or infrequent long-distance dispersal of adult fishes inhabiting the northern South China Sea. Short-distance dispersal is possibly hampered by coral distribution along the coastal and shelf waters off Vietnam (Figure 1a), as adult *Pennahia* prefer sandy or muddy bottoms. The South China Sea Warm Current in the northern South China Sea might also impact the dispersal of pelagic larvae. According to Tuuli (2011) [39], the spawning season for females and males of *P. anea* in the northern South China Sea was observed from March to June. During this period, the northward South China Sea Warm Current near the Hainan region might obstruct most larval dispersal toward the southern South China Sea [86]. 

As discussed above, the formation of the Sunda Shelf barrier may limit population connectivity between Indian and Pacific populations. Following the submergence of Sundaland, the rising of sea level may have allowed the re-colonization of *P. anea* L2 from the South China Sea into the eastern Indian Ocean (Malacca Strait). This re-colonization has been leading to the broad scale genetic homogeneity observed across the South China Sea and eastern Indian Ocean in most *P. anea* L2 populations. Such broad-scale genetic homogeneity has also been reported in some marine fishes *Naso brevirostris*, *N. unicornis* and *N. vlamingii* [87]; *Gymnothorax undulatus* and *G. flavimarginatus* [88]; *Nemipterus japonicus* [32]; *Decapterus maruadsi* [85]. Nevertheless, the observed genetic homogeneity of *P. anea* L2 cannot be claimed with certainty to be a single panmictic population for the species because of significant structuring of the HAI population relative to other populations (see above). 

The pattern of restricted distribution in the eastern Indian Ocean and phylogeographic/population genetic breaks observed in *P. anea* and *P. ovata* have also gained our attention (Figure 1b and Figure 3). Why is the distribution of *P. anea* L1 not widespread as observed in *P. anea* L2? We postulate that the effect of ocean currents in the Malacca Strait may be one of the plausible reasons. According to several previous studies [1,89,90], the Malacca Strait’s currents flow mostly northward toward the Andaman Sea. These northward currents may obstruct the dispersal of pelagic larvae from north to south within the Malacca Strait, and eventually from the Indian Ocean to the South China Sea. Another plausible reason is that the rocky bottom and coral distribution in the Andaman Sea and around the southern part of the Malacca Strait including Singapore may further hamper the migration of adult fishes between the Bay of Bengal and Malacca Strait, and between the Malacca Strait and South China Sea. Such an effect may cause highly localized or high self-recruitment of fish from these regions [89].

Overall, shared historical factors such as climatic and oceanographic changes, formation of the Sunda Shelf barrier, together with contemporary ocean currents and species habitat preferences, all have played important roles, yet at different time scales, in driving the diversification and shaping contemporary phylogeographic patterns of the *Pennahia* species investigated in this study. More empirical studies are still necessary to draw a general conclusion about the evolutionary processes that contribute to the nature of the biodiversity in marine species seen today in SEA waters.

## Figures and Tables

**Table 1:** Descriptive statistics of the sequence datasets used in this study (outgroup sequences excluded). Locus abbreviations: *cytb*, *cytochrome b*; *COI*, *cytochrome oxidase subunit I*; *RAG1*, exon 3 of recombination activating gene 1 and combined gene (*cytb*, *COI* and *RAG1*).

Statistics	Locus			
	*cytb* (all species/*P. anea/P. ovata/P. macrocephalus/P. argentata/P. pawak*)	*COI* (all selected samples)	*RAG1* (all selected samples)	Combined gene (all selected samples)
No. sequence	330/152/53/111/8/6	22	23	23
Length of fragment (bp)	1113/1017/1017/1113/1137/1104	618	1472	3203
No. parsimony-informative sites	342/74/29/60/27/0	157	107	583
No. variable sites (in %)	368 (33.1%)/135 (13.3%)/58 (5.7%)/105 (9.4%)/38 (3.3%)/9 (0.8%)	175 (28.3%)	122 (8.3%)	631 (19.7%)

**Table 2:** Summary of *cytb* gene molecular diversity and statistical tests for *P. anea*, *P. ovata* and *P. macrocephalus*. Two lineages were recognized within *P. anea* which are labelled as lineages L1 and L2. Number of individuals (*n*), number of haplotypes (*nh*), haplotype diversity (*h*) and nucleotide diversity (*π*) are presented. Bold values indicate significance at *p* < 0.05. ** actual total number of haplotypes in all samples. For location abbreviation see Figure 1.

Species	Population	*n*	*nh*	*h*	*π*	Tajima’s D	Fu’s Fs
*P. anea* L1							
	ADM	16	10	0.8667	0.0020	**−2.1986**	**−5.7904**
	NMS L1	11	8	0.8909	0.0027	**−1.9260**	**−3.3664**
	CMS L1	9	8	0.9722	0.0034	−1.5903	**−3.8665**
	All samples	36	23 **	0.8920	0.0025	**−2.4664**	**−21.4200**
*P. anea* L2							
	NMS L2	36	28	0.9857	0.0085	−0.7953	**−13.7489**
	CMS L2	19	17	0.9825	0.0084	−0.7899	**−7.3497**
	EPM	20	16	0.9737	0.0066	−1.2525	**−6.2285**
	GoT	12	11	0.9848	0.0070	−1.2807	**−4.0468**
	HAI	20	14	0.9158	0.0018	**−2.2030**	**−12.5861**
	TWS	5	3	0.7000	0.0012	−1.0485	−0.1859
	All samples	112	73 **	0.9760	0.0071	**−1.8848**	**−24.8560**
*P. anea* L2i		82	58	0.9681	0.0030	**−2.5560**	**−26.4080**
*P. anea* L2ii		22	9	0.8268	0.0026	−1.4757	−1.9738
*P. anea* L2iii		8	6	0.8929	0.0025	**−1.7415**	−2.0500
*P. ovata*							
	BoB	12	7	0.8636	0.0047	0.1259	−0.1384
	NMS	41	26	0.9354	0.0044	**−2.0022**	**−16.9494**
	All samples	53	33 **	0.9550	0.0089	−1.0051	**−12.6100**
*P. macrocephalus*							
	EPM	10	10	1.0000	0.0068	−1.2504	**−4.3504**
	HAI	14	13	0.9890	0.0085	−1.3133	**−4.4778**
	TWS	65	47	0.9856	0.0088	−1.4130	**−24.6058**
	ETW	22	19	0.9870	0.0072	**−1.7346**	**−8.7578**
	All samples	111	79 **	0.9890	0.0084	**−1.7253**	**−24.5060**
*P. macrocephalus* i		77	53	0.9809	0.0053	**−2.0149**	**−25.2652**
*P. macrocephalus* ii		9	8	0.9722	0.0038	−1.5388	**−3.1764**
*P. macrocephalus* iii		25	18	0.9667	0.0044	**−1.5800**	**−8.9588**

**Table 3:** Population pairwise comparisons, Ф_ST_ of *P. anea* lineage L1 (**a**), *P. anea* lineage L2 (**b**), *P. ovata* (**c**) and *P. macrocephalus* (**d**) at *cytb* gene. Bold value indicates significant value after FDR adjustment (*p* < 0.05).

(**a**)						
	**ADM**	**NMS L1**	**CMS L1**			
**ADM**						
**NMS L1**	0.0013					
**CMS L1**	0.0232	0.0000				
(**b**)						
	**NMS L2**	**CMS L2**	**EPM**	**GoT**	**HAI**	**TWS**
**NMS L2**						
**CMS L2**	0.0443					
**EPM**	**0.1044**	0.0000				
**GoT**	0.0972	0.0000	0.0000			
**HAI**	**0.2765**	**0.1219**	**0.0695**	**0.0667**		
**TWS**	0.1923	0.0197	0.0000	0.0000	0.0000	
(**c**)						
	**BoB**	**NMS**				
**BoB**						
**NMS**	**0.5000**					
(**d**)						
	**EPM**	**HAI**	**TWS**	**ETW**		
**EPM**						
**HAI**	0.0138					
**TWS**	0.0609	0.0150				
**ETW**	0.0042	0.0000	0.0409			

**Table 4:** AMOVA result for hierarchical genetic subdivision for the percentage of variation and fixation indices (Ф_ST_, Ф_SC_ and Ф_CT_) of *P. anea* and *P. macrocephalus* at *cytb* gene. Groups are assigned in the parenthesis. Bold value indicates significant value (*p* < 0.05).

Group	Ф_ST_	Ф_SC_	Ф_CT_	Among Groups (%)	Among Populations within Groups (%)	Within Populations (%)
***P. anea***						
**Based on the phylogenetic inference** L1 [ADM, NMS L1, CMS L1] L2 [NMS L2, CMS L2, EPM, GoT, HAI, TWS]	**0.8551**	**0.0921**	**0.8404**	84.04	1.47	14.49
**Based on the ocean region (L2 only)** EIO [NMS L2, CMS L2] SCS [EPM, GoT, HAI, TWS]	**0.1405**	0.0207	0.1224	12.23	1.82	85.95
**Based on pairwise Φ_ST_ (L2 only)** Group 1 [NMS L2, CMS L2, EPM, GoT, TWS] Group 2 [HAI]	**0.1396**	**0.0749**	0.0700	7.00	6.96	86.04
***P. macrocephalus***						
**Based on ecoregion following Spalding et al. (2007) [4]** **3 ecoregions** southern SCS [EPM] northern SCS [HAI] northeastern SCS [TWS, ETW]	**0.0240**	**0.0418**	0.000	−1.86	4.26	97.60
**Based on pairwise Φ_ST_** Group 1 [EPM] Group 2 [HAI, ETW] Group 3 [TWS]	**0.0352**	0.0182	0.0172	1.74	1.79	96.48

EIO = Eastern Indian Ocean; SCS = South China Sea.

## Data Availability

The datasets presented in this study can be found in online repositories. The names of the repository/repositories and accession number(s) can be found below: https://www.ncbi.nlm.nih.gov/genbank/, MN746952-MN841734.

## Supplementary Materials

The following are available online at https://www.mdpi.com/article/10.3390/genes12121926/s1, Table S1: Sample size by species and locality. Species arranged by alphabetical order; localities arranged from West to East. Two lineages were recognized within *P. anea*, and coined lineage L1 and lineage L2. Colours represent the nine populations considered in this paper (see Figure 1a). Kota Kinabalu, Tawau and Singapore were omitted for population consideration due to too low number of samples per site. N is number of samples. Table S2: Taxa, gene, voucher number, GenBank accession number and the sample localities of representative species. In parenthesis is their assigned population. Samples ID of P. anea lineage L1 are written in Italic. Table S3: Representative for each Pennahia species used in estimating the divergence time with their assigned ocean region. In parenthesis is the sampling location. Table S4: Primers used to amplify cytb, COI and RAG1 genes of Pennahia species in this study. Table S5: Mean genetic p-distances among populations, and between different lineages and species of Pennahia species based on the reconstructed cytb tree. Figure S1: Pennahia maximum likelihood phylogenetic tree based on combined (multi-gene) dataset. Branch length is proportional to the inferred number of nucleotide substitutions. Numbers at nodes represent RAxML bootstrap values in percentages and posterior probabilities of BEAST 2 Bayesian inference. Values < 50% or probabilities < 0.50 are not shown. * Indicates bootstrap values ≥ 95% and posterior probabilities ≥ 0.95. Tree was rooted using *Chrysochir aureus* and *Megalonibea fusca*.
