# Supplementary material for: Comparative Phylogeography and Phylogeny of Pennah Croakers (Teleostei: Sciaenidae) in Southeast Asian Waters
Source: Genes (Basel). 2021 Nov 29;12(12):1926. doi: 10.3390/genes12121926 (PMC8701226; doi:10.3390/genes12121926)
Supplement: Supplementary file 1 [file genes-12-01926-s001.zip › genes-1475515-SI.pdf]

Table S1. Sample size by species and locality. Species arranged by alphabetical order; localities arranged from West to East. Two lineages were recognized within *P. anea*, and coined lineage L1 and lineage L2. Colours represent the nine populations considered in this paper (see Figure 1a). Kota Kinabalu, Tawau and Singapore were omitted for population consideration due to too low number of samples per site. *N* is number of samples.

Species/Lineage	Sampling locality																								*N*
	Eastern Indian Ocean							South China Sea																	
	1	2	3	4	5	6	7	8	9	10	11	12	13	14	15	16	17	18	19	20	21	22	23	24	
P. anea L1		16	9	1		1	9																		36
P. anea L2			24	8		4	19	2	10	13	7	1	1	19	1	2		5							116
P. macrocephalus										2	8			14			19	15	1	7	4	19	14	8	111
P. ovata	12		14	3	22	2																			53

1. Riaz, Uddin, Chittagong; 2. Ranong; 3. Kuala Perlis; 4. Penang; 5. Bagan Panchor, Perak; 6. Lumut, Perak; 7. Selangor; 8. Pran Buri; 9. Klong Wan; 10. Kota Bharu, Kelantan; 11. Pulau Kambing, Terengganu; 12. Singapore; 13. QuangYen Town, Vietnam; 14. Hainan; 15. Kota Kinabalu, Sabah; 16. Tawau, Sabah; 17. Penghu; 18. Wuchi, Taiwan; 19. Yunlin, Taiwan; 20. Chaiyi, Taiwan; 21. Anping, Taiwan; 22. Pingtung, Taiwan; 23. Ilan, Taiwan; 24. Taitung, Taiwan

Bay of Bengal (BoB)

Andaman Sea (ADM)

Northern Malacca Strait (NMC)

Central Malacca Strait (CMS)

Gulf of Thailand (GoT)

East Peninsular Malaysia (EPM)

Hai (HAI)

Taiwan Strait (TWS)

East Taiwan (ETW)

Table S2. Taxa, gene, voucher number, GenBank accession number and the sample localities of representative species. In parenthesis is their assigned population. Samples ID of *P. anea* lineage L1 are written in *Italic*.

Species	Sample ID	Voucher No.	Sample site	GenBank Accession Number		
				**Cyt *b***	***COI***	***RAG1***
Pennahia anea	WJC7637	NTUM16446	Ranong, Thailand (ADM)	MN746952	-	-
	WJC7638	-	Ranong, Thailand (ADM)	MN746953	-	-
	WJC7639	-	Ranong, Thailand (ADM)	MN746954	-	-
	WJC7640	-	Ranong, Thailand (ADM)	MN746955	-	-
	WJC7641	-	Ranong, Thailand (ADM)	MN746956	-	-
	WJC7642	-	Ranong, Thailand (ADM)	MN746957	-	-
	WJC7643	-	Ranong, Thailand (ADM)	MN746958	-	-
	WJC7644	-	Ranong, Thailand (ADM)	MN746959	-	-
	WJC7645	-	Ranong, Thailand (ADM)	MN746960	-	-
	WJC7646	-	Ranong, Thailand (ADM)	MN746961	-	-
	WJC7647	-	Ranong, Thailand (ADM)	MN746962	-	-
	WJC7648	-	Ranong, Thailand (ADM)	MN746963	-	-
	WJC7649	-	Ranong, Thailand (ADM)	MN746964	-	-
	WJC7650	-	Ranong, Thailand (ADM)	MN746965	-	-
	WJC7651	-	Ranong, Thailand (ADM)	MN746966	MT778775	MT778779
	WJC7775	NTUM16446	Ranong, Thailand (ADM)	MN746969	-	-
	May128	-	Kuala Perlis, Perlis, Malaysia (NMS)	MN746835	-	-
	May133	-	Kuala Perlis, Perlis, Malaysia (NMS)	MN746840	MT778776	MT778780
	May140	-	Kuala Perlis, Perlis, Malaysia (NMS)	MN746846	-	-
	May146	-	Kuala Perlis, Perlis, Malaysia (NMS)	MN746850	-	-
	May149	-	Kuala Perlis, Perlis, Malaysia (NMS)	MN746852	-	-
	May154	-	Kuala Perlis, Perlis, Malaysia (NMS)	MN746856	-	-
	May156	-	Kuala Perlis, Perlis, Malaysia (NMS)	MN746858	-	-
	May158	-	Kuala Perlis, Perlis, Malaysia (NMS)	MN746860	-	-
	May159	-	Kuala Perlis, Perlis, Malaysia (NMS)	MN746861	-	-
	May169	-	Penang, Malaysia (NMS)	MN746868	-	-
	May101	-	Lumut, Perak, Malaysia (NMS)	MN746828	-	-

Species	Sample ID	Voucher No.	Sample site	GenBank Accession Number		
				Cyt *b*	*COI*	*RAG1*
	May576	-	Selangor, Malaysia (CMS)	MN746914	-	-
	May577	-	Selangor, Malaysia (CMS)	MN746915	-	-
	May580	-	Selangor, Malaysia (CMS)	MN746916	MT778777	MT778781
	May418	-	Selangor, Malaysia (CMS)	MN746883	-	-
	May420	-	Selangor, Malaysia (CMS)	MN746885	-	-
	May429	-	Selangor, Malaysia (CMS)	MN746893	-	-
	May431	-	Selangor, Malaysia (CMS)	MN746895	-	-
	May434	-	Selangor, Malaysia (CMS)	MN746898	-	-
	May435	-	Selangor, Malaysia (CMS)	MN746899	-	-
	May070	-	Kuala Perlis, Perlis, Malaysia (NMS)	MN746827	-	-
	May126	-	Kuala Perlis, Perlis, Malaysia (NMS)	MN746833	KX778049	KX777828
	May127	-	Kuala Perlis, Perlis, Malaysia (NMS)	MN746834	-	-
	May129	-	Kuala Perlis, Perlis, Malaysia (NMS)	MN746836	-	-
	May130	-	Kuala Perlis, Perlis, Malaysia (NMS)	MN746837	-	-
	May131	-	Kuala Perlis, Perlis, Malaysia (NMS)	MN746838	-	-
	May132	-	Kuala Perlis, Perlis, Malaysia (NMS)	MN746839	-	-
	May135	-	Kuala Perlis, Perlis, Malaysia (NMS)	MN746841	-	-
	May136	-	Kuala Perlis, Perlis, Malaysia (NMS)	MN746842	-	-
	May137	-	Kuala Perlis, Perlis, Malaysia (NMS)	MN746843	-	-
	May138	-	Kuala Perlis, Perlis, Malaysia (NMS)	MN746844	-	-
	May139	-	Kuala Perlis, Perlis, Malaysia (NMS)	MN746845	-	-
	May143	-	Kuala Perlis, Perlis, Malaysia (NMS)	MN746847	-	-
	May144	-	Kuala Perlis, Perlis, Malaysia (NMS)	MN746848	-	-
	May145	-	Kuala Perlis, Perlis, Malaysia (NMS)	MN746849	-	-
	May147	-	Kuala Perlis, Perlis, Malaysia (NMS)	MN746851	-	-
	May150	-	Kuala Perlis, Perlis, Malaysia (NMS)	MN746853	-	-
	May151	-	Kuala Perlis, Perlis, Malaysia (NMS)	MN746854	-	-
	May153	-	Kuala Perlis, Perlis, Malaysia (NMS)	MN746855	-	-
	May155	-	Kuala Perlis, Perlis, Malaysia (NMS)	MN746857	-	-
	May157	-	Kuala Perlis, Perlis, Malaysia (NMS)	MN746859	-	-

Species	Sample ID	Voucher No.	Sample site	GenBank Accession Number		
				Cyt b	***COI***	***RAG1***
	May161	-	Kuala Perlis, Perlis, Malaysia (NMS)	MN746862	-	-
	May163	-	Kuala Perlis, Perlis, Malaysia (NMS)	MN746863	-	-
	May164	-	Kuala Perlis, Perlis, Malaysia (NMS)	MN746864	-	-
	May166	-	Penang, Malaysia (NMS)	MN746865	-	-
	May167	-	Penang, Malaysia (NMS)	MN746866	-	-
	May168	-	Penang, Malaysia (NMS)	MN746867	-	-
	May170	-	Penang, Malaysia (NMS)	MN746869	-	-
	May787	-	Penang, Malaysia (NMS)	MN746918	-	-
	May788	-	Penang, Malaysia (NMS)	MN746919	-	-
	May789	-	Penang, Malaysia (NMS)	MN746920	-	-
	May790	-	Penang, Malaysia (NMS)	MN746921	-	-
	May102	-	Lumut, Perak, Malaysia (NMS)	MN746829	-	-
	May103	-	Lumut, Perak, Malaysia (NMS)	MN746831	-	-
	May104	-	Lumut, Perak, Malaysia (NMS)	MN746830	-	-
	May105	-	Lumut, Perak, Malaysia (NMS)	MN746832	KX778048	KX777827
	May568	-	Selangor, Malaysia (CMS)	MN746907	-	-
	May569	-	Selangor, Malaysia (CMS)	MN746908	-	-
	May570	-	Selangor, Malaysia (CMS)	MN746909	-	-
	May571	-	Selangor, Malaysia (CMS)	MN746910	-	-
	May572	-	Selangor, Malaysia (CMS)	MN746911	-	-
	May573	-	Selangor, Malaysia (CMS)	MN746912	-	-
	May574	-	Selangor, Malaysia (CMS)	MN746913	-	-
	May578	-	Selangor, Malaysia (CMS)	MN746917	-	-
	May419	-	Selangor, Malaysia (CMS)	MN746884	-	-
	May421	-	Selangor, Malaysia (CMS)	MN746886	-	-
	May422	-	Selangor, Malaysia (CMS)	MN746887	-	-
	May423	-	Selangor, Malaysia (CMS)	MN746888	-	-
	May425	-	Selangor, Malaysia (CMS)	MN746889	-	-
	May426	-	Selangor, Malaysia (CMS)	MN746890	-	-
	May427	-	Selangor, Malaysia (CMS)	MN746891	-	-

Species	Sample ID	Voucher No.	Sample site	GenBank Accession Number		
				Cyt b	***COI***	***RAG1***
	May428	-	Selangor, Malaysia (CMS)	MN746892	-	-
	May430	-	Selangor, Malaysia (CMS)	MN746894	-	-
	May432	-	Selangor, Malaysia (CMS)	MN746896	-	-
	May433	-	Selangor, Malaysia (CMS)	MN746897	-	-
	May197	-	Kota Bharu, Kelantan, Malaysia (EPM)	MN746870	-	-
	May198	-	Kota Bharu, Kelantan, Malaysia (EPM)	MN746871	-	-
	May199	-	Kota Bharu, Kelantan, Malaysia (EPM)	MN746872	-	-
	May200	-	Kota Bharu, Kelantan, Malaysia (EPM)	MN746873	-	-
	May201	-	Kota Bharu, Kelantan, Malaysia (EPM)	MN746874	-	-
	May202	-	Kota Bharu, Kelantan, Malaysia (EPM)	MN746875	MT778778	MT778782
	May203	-	Kota Bharu, Kelantan, Malaysia (EPM)	MN746876	-	-
	May204	-	Kota Bharu, Kelantan, Malaysia (EPM)	MN746877	-	-
	May205	-	Kota Bharu, Kelantan, Malaysia (EPM)	MN746878	-	-
	May206	-	Kota Bharu, Kelantan, Malaysia (EPM)	MN746879	-	-
	May207	-	Kota Bharu, Kelantan, Malaysia (EPM)	MN746880	-	-
	May208	-	Kota Bharu, Kelantan, Malaysia (EPM)	MN746881	-	-
	May209	-	Kota Bharu, Kelantan, Malaysia (EPM)	MN746882	-	-
	May487	-	Pualu Kambing, Terangganu, Malaysia (EPM)	MN746900	-	-
	May488	-	Pualu Kambing, Terangganu, Malaysia (EPM)	MN746901	-	-
	May489	-	Pualu Kambing, Terangganu, Malaysia (EPM)	MN746902	-	-
	May490	-	Pualu Kambing, Terangganu, Malaysia (EPM)	MN746903	-	-
	May491	-	Pualu Kambing, Terangganu, Malaysia (EPM)	MN746904	-	-
	May492	-	Pualu Kambing, Terangganu, Malaysia (EPM)	MN746905	-	-
	May493	-	Pualu Kambing, Terangganu, Malaysia (EPM)	MN746906	-	-
	WJC7565	-	Klong wan, Thailand (GoT)	MN746942	-	-
	WJC7566	-	Klong wan, Thailand (GoT)	MN746943	-	-
	WJC7567	-	Klong wan, Thailand (GoT)	MN746944	-	-
	WJC7568	-	Klong wan, Thailand (GoT)	MN746945	-	-
	WJC7569	-	Klong wan, Thailand (GoT)	MN746946	-	-
	WJC7570	-	Klong wan, Thailand (GoT)	MN746947	-	-

Species	Sample ID	Voucher No.	Sample site	GenBank Accession Number		
				Cyt b	*COI*	*RAG1*
	WJC7571	-	Klong wan, Thailand (GoT)	MN746948	-	-
	WJC7572	-	Klong wan, Thailand (GoT)	MN746949	-	-
	WJC7573	-	Klong wan, Thailand (GoT)	MN746950	-	-
	WJC7574	-	Klong wan, Thailand (GoT)	MN746951	-	-
	WJC7713	-	Pran Buri, Thailand (GoT)	MN746967	-	-
	WJC7714	-	Pran Buri, Thailand (GoT)	MN746968	-	-
	WJC1750	NTUM11610	QuangYen Town, Vietnam (HAI)	MN746922	KX778050	KX777829
	WJC1763	-	Hainan, China (HAI)	MN746923	KX778051	KX777830
	WJC1784	NTUM11623	Hainan, China (HAI)	MN746924	KX778052	KX777831
	WJC4753	-	Hainan, China (HAI)	MN746941	-	-
	WJC1811	-	Hainan, China (HAI)	MN746925	-	-
	WJC4598	NTUM11717	Hainan, China (HAI)	MN746926	-	-
	WJC4599	-	Hainan, China (HAI)	MN746927	-	-
	WJC4600	-	Hainan, China (HAI)	MN746928	-	-
	WJC4601	-	Hainan, China (HAI)	MN746929	-	-
	WJC4602	-	Hainan, China (HAI)	MN746930	-	-
	WJC4603	NTUM11719	Hainan, China (HAI)	MN746931	-	-
	WJC4604	-	Hainan, China (HAI)	MN746932	-	-
	WJC4605	-	Hainan, China (HAI)	MN746933	-	-
	WJC4606	-	Hainan, China (HAI)	MN746934	-	-
	WJC4607	-	Hainan, China (HAI)	MN746935	-	-
	WJC4608	-	Hainan, China (HAI)	MN746936	-	-
	WJC4609	-	Hainan, China (HAI)	MN746937	-	-
	WJC4610	-	Hainan, China (HAI)	MN746938	-	-
	WJC4648	-	Hainan, China (HAI)	MN746939	-	-
	WJC4649	-	Hainan, China (HAI)	MN746940	-	-
	WJC1939	-	Pulau Ubin, Singapore	MT778784	KX778053	KX777832
	WJC1956	NTUM11644	Wuchi, Taiwan (TWS)	MT778785	-	-
	WJC2595	NTUM11685	Wuchi, Taiwan (TWS)	MT778786	-	-
	WJC2596	NTUM11685	Wuchi, Taiwan (TWS)	MT778787	-	-

Species	Sample ID	Voucher No.	Sample site	GenBank Accession Number Cyt b	COI	RAG1
	WJC2597	NTUM11685	Wuchi, Taiwan (TWS)	MT778788	KX778054	KX777833
	WJC3506	NTUM11704	Wuchi, Taiwan (TWS)	MT778789	-	-
	WJC5285	NTUM11732	Kota Kinabalu, Sabah, Malaysia	MT778790	KX778055	KX777834
	WJC5573	-	Tawau, Sabah, Malaysia	MT778791	-	-
	WJC5574	-	Tawau, Sabah, Malaysia	MT778792	-	-
P. argentata	CN172	-	Penghu, Taiwan	KP722669	KP722758	KP722941
	CN258	-	Longkou port, Shandong, China	MT778793	KX778057	KX777836
	CN299	-	HK Aberdeen Fish Market, Hong Kong	MT778794	KX778058	KX777837
	CN592	-	Xiamen, China	MT778795	KX778059	KX777838
	WJC0880	NTUM11551	Wuchi, Taiwan	MT778796	KX778061	KX777840
	WJC0984	NTUM11560	Jagalchi, Korea	MT778797	KX778062	KX777841
	WJC6398	-	Off Hirono, Fukushima, Japan	MT778798	KX778069	KX777848
	WJC6439	NTUM11740	Tokyo Bay, Japan	MT778799	KX778070	KX777849
P. macrocephalus	May185	-	Kota Bharu, Kelantan, Malaysia (EPM)	MT778664	-	-
	May186	-	Kota Bharu, Kelantan, Malaysia (EPM)	MT778665	KX778073	KX777852
	May447	-	Pualu Kambing, Terangganu, Malaysia (EPM)	MT778666	KX778074	KX777853
	May448	-	Pualu Kambing, Terangganu, Malaysia (EPM)	MT778667	-	-
	May449	-	Pualu Kambing, Terangganu, Malaysia (EPM)	MT778668	-	-
	May450	-	Pualu Kambing, Terangganu, Malaysia (EPM)	MT778669	-	-
	May451	-	Pualu Kambing, Terangganu, Malaysia (EPM)	MT778670	-	-
	May452	-	Pualu Kambing, Terangganu, Malaysia (EPM)	MT778671	-	-
	May453	-	Pualu Kambing, Terangganu, Malaysia (EPM)	MT778672	-	-
	May454	-	Pualu Kambing, Terangganu, Malaysia (EPM)	MT778673	-	-
	WJC1786	NTUM11625	Hainan, China (HAI)	MT778674	KX778080	KX777859
	WJC1796	NTUM11628	Hainan, China (HAI)	MT778675	-	-
	WJC4754	NTUM11726	Hainan, China (HAI)	MT778676	-	-
	WJC4755	-	Hainan, China (HAI)	MT778677	-	-
	WJC4756	-	Hainan, China (HAI)	MT778678	-	-

Species	Sample ID	Voucher No.	Sample site	GenBank Accession Number		
				Cyt b	*COI*	*RAG1*
	WJC4757	-	Hainan, China (HAI)	MT778679	-	-
	WJC4758	-	Hainan, China (HAI)	MT778680	-	-
	WJC4759	-	Hainan, China (HAI)	MT778681	-	-
	WJC4760	-	Hainan, China (HAI)	MT778682	-	-
	WJC4761	-	Hainan, China (HAI)	MT778683	-	-
	WJC4762	-	Hainan, China (HAI)	MT778684	-	-
	WJC4763	-	Hainan, China (HAI)	MT778685	-	-
	WJC4764	-	Hainan, China (HAI)	MT778686	-	-
	WJC4765	-	Hainan, China (HAI)	MT778687	-	-
	WJC0918	NTUM11554	Ilan, Taiwan (ETW)	MT778688	KX778079	KX777858
	WJC0919	NTUM11554	Ilan, Taiwan (ETW)	MT778689	-	-
	WJC0920	NTUM11554	Ilan, Taiwan (ETW)	MT778690	-	-
	WJC0921	NTUM11554	Ilan, Taiwan (ETW)	MT778691	-	-
	WJC4882	NTUM11727	Ilan, Taiwan (ETW)	MT778692	-	-
	WJC4883	NTUM11727	Ilan, Taiwan (ETW)	MT778693	-	-
	WJC4884	NTUM11727	Ilan, Taiwan (ETW)	MT778694	-	-
	WJC4885	NTUM11727	Ilan, Taiwan (ETW)	MT778695	-	-
	WJC4886	NTUM11727	Ilan, Taiwan (ETW)	MT778696	-	-
	WJC4887	NTUM11727	Ilan, Taiwan (ETW)	MT778697	-	-
	WJC4888	NTUM11727	Ilan, Taiwan (ETW)	MT778698	-	-
	WJC4889	NTUM11727	Ilan, Taiwan (ETW)	MT778699	-	-
	WJC4890	NTUM11727	Ilan, Taiwan (ETW)	MT778700	-	-
	WJC4891	NTUM11727	Ilan, Taiwan (ETW)	MT778701	-	-
	WJC4558	NTUM11707	Taitung, Taiwan (ETW)	MT778702	KX778081	KX777860
	WJC4559	NTUM11707	Taitung, Taiwan (ETW)	MT778703	-	-
	WJC4560	NTUM11707	Taitung, Taiwan (ETW)	MT778704	-	-
	WJC4561	NTUM11707	Taitung, Taiwan (ETW)	MT778705	-	-
	WJC4562	NTUM11707	Taitung, Taiwan (ETW)	MT778706	-	-
	WJC4563	NTUM11707	Taitung, Taiwan (ETW)	MT778707	-	-
	WJC4564	NTUM11707	Taitung, Taiwan (ETW)	MT778708	-	-

Species	Sample ID	Voucher No.	Sample site	GenBank Accession Number		
				Cyt b	*COI*	*RAG1*
	WJC4565	NTUM11707	Taitung, Taiwan (ETW)	MT778709	-	-
	WJC0666	NTUM11527	Wuchi, Taiwan (TWS)	MT778710	-	-
	WJC0674	NTUM11527	Wuchi, Taiwan (TWS)	MT778711	-	-
	WJC0679	NTUM11536	Wuchi, Taiwan (TWS)	MT778712	KX778077	KX777856
	WJC0680	NTUM11536	Wuchi, Taiwan (TWS)	MT778713	-	-
	WJC0687	NTUM11527	Wuchi, Taiwan (TWS)	MT778714	-	-
	WJC0728	NTUM11542	Wuchi, Taiwan (TWS)	MT778715	-	-
	WJC0729	NTUM11542	Wuchi, Taiwan (TWS)	MT778716	-	-
	WJC0882	NTUM11552	Wuchi, Taiwan (TWS)	MT778717	-	-
	WJC0883	NTUM11552	Wuchi, Taiwan (TWS)	MT778718	-	-
	WJC1035	NTUM11567	Wuchi, Taiwan (TWS)	MT778719	-	-
	WJC1036	NTUM11567	Wuchi, Taiwan (TWS)	MT778720	-	-
	WJC1072	NTUM11571	Wuchi, Taiwan (TWS)	MT778721	-	-
	WJC1073	NTUM11571	Wuchi, Taiwan (TWS)	MT778722	-	-
	WJC2136	NTUM11678	Wuchi, Taiwan (TWS)	MT778723	-	-
	WJC2137	NTUM11678	Wuchi, Taiwan (TWS)	MT778724	-	-
	WJC1112	-	Yunlin, Taiwan (TWS)	MT778725	-	-
	WJC5087	NTUM11729	Penghu, Taiwan (TWS)	MT778726	-	-
	WJC5088	NTUM11729	Penghu, Taiwan (TWS)	MT778727	KX778082	KX777861
	WJC5089	NTUM11729	Penghu, Taiwan (TWS)	MT778728	-	-
	WJC5090	NTUM11729	Penghu, Taiwan (TWS)	MT778729	-	-
	WJC5091	NTUM11729	Penghu, Taiwan (TWS)	MT778730	-	-
	WJC5092	NTUM11729	Penghu, Taiwan (TWS)	MT778731	-	-
	WJC5093	NTUM11729	Penghu, Taiwan (TWS)	MT778732	-	-
	WJC5094	NTUM11729	Penghu, Taiwan (TWS)	MT778733	-	-
	WJC5095	NTUM11729	Penghu, Taiwan (TWS)	MT778734	-	-
	WJC5096	-	Penghu, Taiwan (TWS)	MT778735	-	-
	WJC5098	-	Penghu, Taiwan (TWS)	MT778736	-	-
	WJC5099	-	Penghu, Taiwan (TWS)	MT778737	-	-
	WJC5100	-	Penghu, Taiwan (TWS)	MT778738	-	-

				GenBank Accession Number		
**Species**	**Sample ID**	**Voucher No.**	**Sample site**	***Cyt b***	***COI***	***RAG1***
	WJC5101	-	Penghu, Taiwan (TWS)	MT778739	-	-
	WJC5102	-	Penghu, Taiwan (TWS)	MT778740	-	-
	WJC5103	-	Penghu, Taiwan (TWS)	MT778741	-	-
	WJC5104	-	Penghu, Taiwan (TWS)	MT778742	-	-
	WJC5138	NTUM11747	Penghu, Taiwan (TWS)	MT778743	-	-
	WJC5139	NTUM11747	Penghu, Taiwan (TWS)	MT778744	-	-
	WJC0427	-	Chaiyi, Taiwan (TWS)	MT778745	-	-
	WJC0658	NTUM11523	Chaiyi, Taiwan (TWS)	MT778746	-	-
	WJC0659	NTUM11523	Chaiyi, Taiwan (TWS)	MT778747	-	-
	WJC0488	NTUM11515	Chaiyi, Taiwan (TWS)	MT778748	KX778076	KX777855
	WJC0491	-	Chaiyi, Taiwan (TWS)	MT778749	-	-
	WJC0651	NTUM11521	Chaiyi, Taiwan (TWS)	MT778750	-	-
	WJC0652	NTUM11521	Chaiyi, Taiwan (TWS)	MT778751	-	-
	WJC0483	NTUM11511	Anping, Taiwan (TWS)	MT778752	KX778075	KX777854
	WJC1567	NTUM11587	Anping, Taiwan (TWS)	MT778753	-	-
	WJC1568	NTUM11587	Anping, Taiwan (TWS)	MT778754	-	-
	WJC1569	NTUM11587	Anping, Taiwan (TWS)	MT778755	-	-
	WJC3531	-	Pingtung, Taiwan (TWS)	MT778756	-	-
	WJC3532	-	Pingtung, Taiwan (TWS)	MT778757	-	-
	WJC3533	-	Pingtung, Taiwan (TWS)	MT778758	-	-
	WJC3534	-	Pingtung, Taiwan (TWS)	MT778759	-	-
	WJC3535	-	Pingtung, Taiwan (TWS)	MT778760	-	-
	WJC3536	-	Pingtung, Taiwan (TWS)	MT778761	-	-
	WJC3537	-	Pingtung, Taiwan (TWS)	MT778762	-	-
	WJC3538	-	Pingtung, Taiwan (TWS)	MT778763	-	-
	WJC3539	-	Pingtung, Taiwan (TWS)	MT778764	-	-
	WJC3540	-	Pingtung, Taiwan (TWS)	MT778765	-	-
	WJC3541	-	Pingtung, Taiwan (TWS)	MT778766	-	-
	WJC3542	-	Pingtung, Taiwan (TWS)	MT778767	-	-
	CN640	-	Pingtung, Taiwan (TWS)	MT778768	-	-

Species	Sample ID	Voucher No.	Sample site	GenBank Accession Number		
				Cyt b	*COI*	*RAG1*
	CN641	-	Pingtung, Taiwan (TWS)	MT778769	-	-
	CN642	-	Pingtung, Taiwan (TWS)	MT778770	-	-
	CN643	-	Pingtung, Taiwan (TWS)	MT778771	-	-
	WJC1643	NTUM11602	Pingtung, Taiwan (TWS)	MT778772	-	-
	WJC1644	NTUM11602	Pingtung, Taiwan (TWS)	MT778773	-	-
	WJC1645	NTUM11602	Pingtung, Taiwan (TWS)	MT778774	-	-
P. ovata	WJC8498	-	Riaz Uddin, Chittagong, Bangladesh (BoB)	MN841741	-	-
	WJC8499	-	Riaz Uddin, Chittagong, Bangladesh (BoB)	MN841742	-	-
	WJC8500	-	Riaz Uddin, Chittagong, Bangladesh (BoB)	MN841743	-	MT778783
	WJC8502	-	Riaz Uddin, Chittagong, Bangladesh (BoB)	MN841744	-	-
	WJC8503	-	Riaz Uddin, Chittagong, Bangladesh (BoB)	MN841745	-	-
	WJC8504	-	Riaz Uddin, Chittagong, Bangladesh (BoB)	MN841746	-	-
	WJC8505	-	Riaz Uddin, Chittagong, Bangladesh (BoB)	MN841747	-	-
	WJC8506	-	Riaz Uddin, Chittagong, Bangladesh (BoB)	MN841748	-	-
	WJC8507	-	Riaz Uddin, Chittagong, Bangladesh (BoB)	MN841749	-	-
	WJC8509	-	Riaz Uddin, Chittagong, Bangladesh (BoB)	MN841750	-	-
	WJC8510	-	Riaz Uddin, Chittagong, Bangladesh (BoB)	MN841751	-	-
	WJC8511	-	Riaz Uddin, Chittagong, Bangladesh (BoB)	MN841752	-	-
	May067	-	Kuala Perlis, Perlis, Malaysia (NMS)	MN841701	-	-
	May068	-	Kuala Perlis, Perlis, Malaysia (NMS)	MN841702	-	-
	May069	-	Kuala Perlis, Perlis, Malaysia (NMS)	MN841703	-	-
	May071	-	Kuala Perlis, Perlis, Malaysia (NMS)	MN841704	-	-
	May072	-	Kuala Perlis, Perlis, Malaysia (NMS)	MN841705	-	-
	May073	-	Kuala Perlis, Perlis, Malaysia (NMS)	MN841706	-	-
	May074	-	Kuala Perlis, Perlis, Malaysia (NMS)	MN841707	-	-
	May075	-	Kuala Perlis, Perlis, Malaysia (NMS)	MN841708	-	-
	May076	-	Kuala Perlis, Perlis, Malaysia (NMS)	MN841709	KX778085	KX777864
	May165	-	Kuala Perlis, Perlis, Malaysia (NMS)	MN841712	-	-
	May682	-	Kuala Perlis, Perlis, Malaysia (NMS)	MN841735	-	-

Species	Sample ID	Voucher No.	Sample site	GenBank Accession Number		
				Cyt b	*COI*	*RAG1*
	May683	-	Kuala Perlis, Perlis, Malaysia (NMS)	MN841736	-	-
	May684	-	Kuala Perlis, Perlis, Malaysia (NMS)	MN841737	-	-
	May685	-	Kuala Perlis, Perlis, Malaysia (NMS)	MN841738	-	-
	CN239	-	Penang, Malaysia (NMS)	MN841700	KX778084	KX777863
	May791	-	Penang, Malaysia (NMS)	MN841739	-	-
	May793	-	Penang, Malaysia (NMS)	MN841740	-	-
	May106	-	Lumut, Perak, Malaysia (NMS)	MN841710	KX778086	KX777865
	May107	-	Lumut, Perak, Malaysia (NMS)	MN841711	-	-
	May605	-	Bagan Panchor, Perak, Malaysia (NMS)	MN841713	-	-
	May606	-	Bagan Panchor, Perak, Malaysia (NMS)	MN841714	-	-
	May607	-	Bagan Panchor, Perak, Malaysia (NMS)	MN841715	-	-
	May608	-	Bagan Panchor, Perak, Malaysia (NMS)	MN841716	-	-
	May609	-	Bagan Panchor, Perak, Malaysia (NMS)	MN841717	-	-
	May611	-	Bagan Panchor, Perak, Malaysia (NMS)	MN841718	-	-
	May613	-	Bagan Panchor, Perak, Malaysia (NMS)	MN841719	-	-
	May615	-	Bagan Panchor, Perak, Malaysia (NMS)	MN841720	-	-
	May617	-	Bagan Panchor, Perak, Malaysia (NMS)	MN841721	-	-
	May618	-	Bagan Panchor, Perak, Malaysia (NMS)	MN841722	-	-
	May620	-	Bagan Panchor, Perak, Malaysia (NMS)	MN841723	-	-
	May621	-	Bagan Panchor, Perak, Malaysia (NMS)	MN841724	-	-
	May622	-	Bagan Panchor, Perak, Malaysia (NMS)	MN841725	-	-
	May624	-	Bagan Panchor, Perak, Malaysia (NMS)	MN841726	-	-
	May626	-	Bagan Panchor, Perak, Malaysia (NMS)	MN841727	-	-
	May627	-	Bagan Panchor, Perak, Malaysia (NMS)	MN841728	-	-
	May628	-	Bagan Panchor, Perak, Malaysia (NMS)	MN841729	-	-
	May629	-	Bagan Panchor, Perak, Malaysia (NMS)	MN841730	-	-
	May630	-	Bagan Panchor, Perak, Malaysia (NMS)	MN841731	-	-
	May631	-	Bagan Panchor, Perak, Malaysia (NMS)	MN841732	-	-
	May632	-	Bagan Panchor, Perak, Malaysia (NMS)	MN841733	-	-
	May633	-	Bagan Panchor, Perak, Malaysia (NMS)	MN841734	-	-

Species	Sample ID	Voucher No.	Sample site	GenBank Accession Number		
				Cyt b	***COI***	***RAG1***
P. pawak	May187	-	Kota Bharu, Kelantan, Malaysia	MT778800	KX778087	KX777866
	WJC1753	NTUM11612	VanDon Town, Vietnam	MT778801	KX778089	KX777868
	WJC0448	NTUM11507	Anping, Taiwan	KP722671	-	-
	WJC1748	NTUM11608	HaLong, Vietnam	MT778802	-	-
*Chrysochir aureus**	WJC0451	NTUM11510		KP722617	KP722708	KP722892
*Megalonibea fusca**	WJC1818	-		KP722646	KP722735	KP722922

*Out-group

Table S3. Representative for each *Pennahia* species used in estimating the divergence time with their assigned ocean region. In parenthesis is the sampling location.

		Region			
**Species**	**Sample ID**	**EIO**	**SCS**	**ECS**	**NWP**
P. anea L1	WJC7651(Ranong, Thailand)	+			
	MAY133 (Kuala Perlis, Malaysia)	+			
	MAY580 (Selangor, Malaysia)	+			
P. anea L2	MAY126 (Kuala Perlis, Malaysia)	+			
	MAY105 (Lumut, Malaysia)	+			
	MAY202 (Kota Bharu, Malaysia)		+		
	WJC1763 (Hainan, China)		+		
	WJC5285 (Kota Kinabalu, Malaysia)		+		
	WJC2597 (Wuchi, Taiwan)		+		
P. argentata	CN172 (Penghu, Taiwan)		+		
	CN299 (Hong Kong, China)		+		
	WJC0984 (Jagalchi, Korea)			+	
	WJC6398 (Fukushima, Japan)				+
	WJC6439 (Tokyo, Japan)				+
P. macrocephalus	MAY447 (Pulau Kambing, Malaysia)		+		
	WJC1786 (Hainan, China)		+		
	WJC0679 (Wuchi, Taiwan)		+		
	CN640 (Pingtung, Taiwan)		+		
	WJC0918 (Ilan, Taiwan)		+		
P. ovata	WJC8500 (Chittagong, Bangladesh)	+			
	MAY106 (Lumut, Malaysia)	+			
P. pawak	MAY187 (Kota Bharu, Malaysia)		+		
	WJC1753 (VanDon, Vietnam)		+		

EIO: East Indian Ocean; SCS: South China Sea; ECS: East China Sea; NWP: northwestern Pacific (Japan)

Table S4. Primers used to amplify *cytb*, *COI* and *RAG1* genes of *Pennahia* species in this study.

Locus	Primer name	Primer sequence (5'-3')	Source
cytb	Pennah 14771F	F: CTAACCACGCACTAGTGGACCT	This study
	MT 14673F	F: TAACCAGGACTAATGGCTTGAAA	Borsa et al., 2013
	Perco 15053F	F: TACAAAGAAACMTGAAACATYGGAGT	Chen, unpubl.
	Sciae 15851R	R: GGTGCTCTGANRCTGAGCTACTA	Lo et al., 2015
COI	CoxI FishF1	F: TCAACCAACCACAAAGACATTGGCAC	Ward et al., 2005
	CoxI FishF2	F: TCGACTAATCATAAAGATATCGGCAC	Ward et al., 2005
	CoxI FishR1	R: TAGACTTCTGGGTGGCCAAAGAATCA	Ward et al., 2005
	CoxI FishR2	R: ACTTCAGGGTGACCGAAGAATCAGAA	Ward et al., 2005
RAG1	R1 2533F	F: CTGAGCTGCAGTCAGTACCATAAGATGT	López et al., 2004
	R1 4090R	R: CTGAGTCCTTGTGAGCTTCCATRAAYTT	López et al., 2004
	R1 4061R	R: AATACTTGGAGGTGTAGAGCCAGT	Chen et al., 2007

Sources

Borsa, P., Hsiao, D.R., Carpenter, K.E., Chen, W.J., 2013. Cranial morphometrics and mitochondrial DNA sequences distinguish cryptic species of the longface emperor (*Lethrinus olivaceus*), an emblematic fish of Indo-West Pacific coral reefs. *C. R. Biol.* 336, 505–514.

Chen, W.J., Ruiz-Carus, R., Ortí, G., 2007. Relationships among four genera of mojarras (Teleostei: Perciformes: Gerreidae) from the western Atlantic and their tentative placement among percomorph fishes. J. Fish Biol. 70, 202–218.

Lo, P.C., Liu, S-H., Chao, N. L., Nunoo, F. K. E., Mok, H.K., Chen, W.J., 2015. A multigene dataset reveals a tropical New World origin and Early Miocene diversification of croakers (Perciformes: Sciaenidae). *Mol. Phylogenet. Evol.* 88, 132–143.

López, J.A., Chen, W.J., Ortí, G., 2004. Esociform phylogeny. *Copeia* 2004, 449–464.

Ward, R.D., Zemlak, T.S., Innes, B.H., Last, P.R., Hebert, P.D.N., 2005. DNA barcoding Australia's fish species. *Phil. Trans. R. Soc. B* 360, 1847–1857.

Table S5. Mean genetic *p*-distances among populations, and between different lineages and species of *Pennahia* species based on the reconstructed *cytb* tree.

		1	2	3	4	5	6	7	8	9	10	11	12	13	14	15	16	17	18	19	20	21
1	*P. anea* ADM																					
2	*P. anea* NMS L1	0.002																				
3	*P. anea* CMS L1	0.003	0.003																			
4	*P. anea* NMS L2	0.038	0.038	0.038																		
5	*P. anea* CMS L2	0.040	0.040	0.041	0.010																	
6	*P. anea* EPM	0.039	0.039	0.040	0.009	0.008																
7	*P. anea* GoT	0.040	0.040	0.040	0.009	0.008	0.007															
8	*P. anea* HAI	0.039	0.040	0.040	0.008	0.006	0.005	0.005														
9	*P. anea* TWS	0.039	0.040	0.040	0.008	0.006	0.005	0.005	0.002													
10	*P. anea* SGP	0.039	0.039	0.039	0.009	0.008	0.006	0.007	0.003	0.003												
11	*P. anea* SBH	0.039	0.039	0.039	0.007	0.006	0.004	0.004	0.001	0.001	0.003											
12	*P. anea* CLS	0.040	0.040	0.040	0.008	0.007	0.005	0.005	0.002	0.002	0.004	0.001										
13	*P. argentata* NWP	0.192	0.191	0.192	0.196	0.197	0.197	0.197	0.197	0.196	0.197	0.197	0.197									
14	*P. argentata* SCS+ECS	0.188	0.188	0.188	0.193	0.193	0.193	0.194	0.194	0.193	0.193	0.193	0.193	0.022								
15	*P. macrocephalus* EPM	0.197	0.197	0.197	0.199	0.199	0.199	0.199	0.199	0.198	0.201	0.198	0.198	0.119	0.117							
16	*P. macrocephalus* HAI	0.196	0.196	0.196	0.197	0.197	0.197	0.197	0.197	0.196	0.199	0.197	0.197	0.117	0.115	0.009						
17	*P. macrocephalus* TWS	0.196	0.196	0.196	0.197	0.197	0.197	0.197	0.197	0.196	0.199	0.197	0.197	0.117	0.114	0.011	0.010					
18	*P. macrocephalus* ETW	0.197	0.197	0.197	0.198	0.198	0.198	0.198	0.198	0.197	0.200	0.198	0.198	0.118	0.115	0.009	0.009	0.010				
19	*P. ovata* BoB	0.164	0.164	0.164	0.160	0.161	0.162	0.161	0.163	0.162	0.165	0.162	0.162	0.174	0.174	0.166	0.166	0.166	0.167			
20	*P. ovata* NMS	0.165	0.165	0.165	0.161	0.163	0.164	0.163	0.165	0.164	0.164	0.164	0.163	0.175	0.175	0.168	0.168	0.168	0.169	0.017		
21	*P. pawak*	0.198	0.198	0.199	0.191	0.192	0.193	0.193	0.194	0.194	0.193	0.193	0.192	0.118	0.113	0.161	0.160	0.160	0.160	0.198	0.197	
22	Outgroup	0.206	0.206	0.206	0.208	0.207	0.208	0.208	0.208	0.208	0.208	0.208	0.209	0.156	0.155	0.161	0.162	0.161	0.162	0.189	0.194	0.173

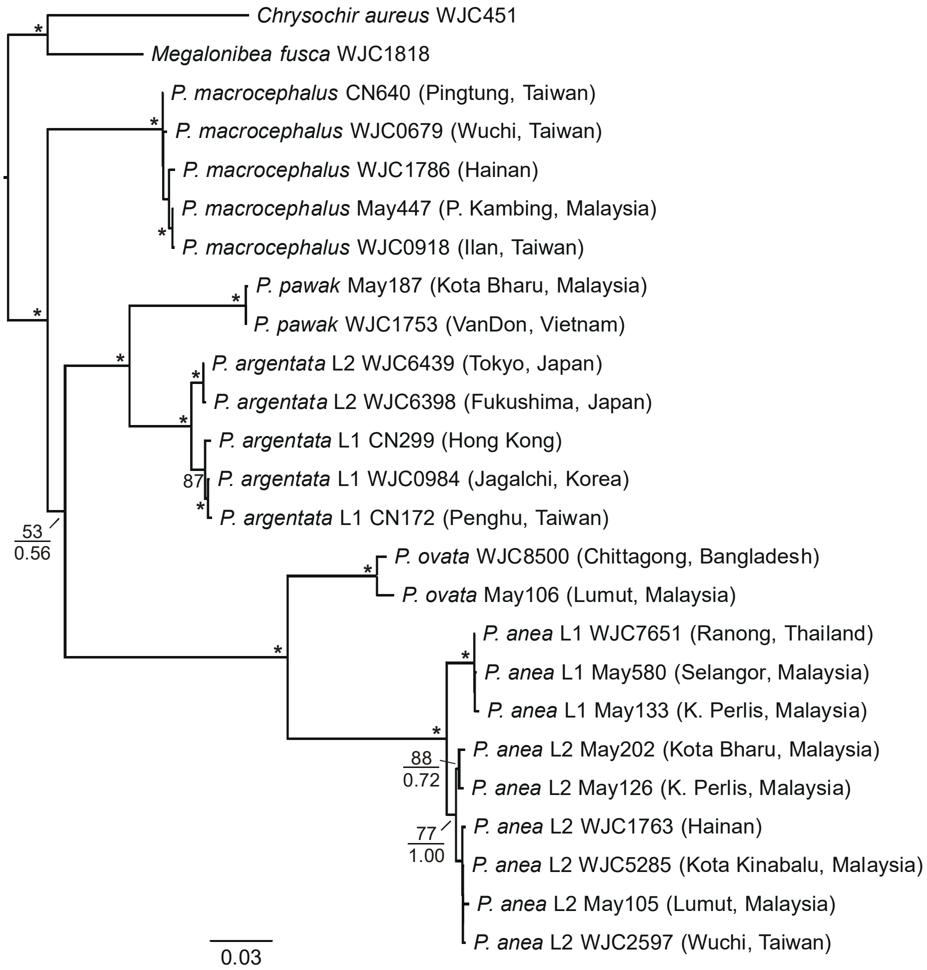

Figure S1. *Pennahia* maximum likelihood phylogenetic tree based on combined (multi-gene) dataset. Branch length is proportional to the inferred number of nucleotide substitutions. Numbers at nodes represent RAxML bootstrap values in percentages and posterior probabilities of BEAST 2 Bayesian inference. Values < 50% or probabilities < 0.50 are not shown. * indicates bootstrap values ≥ 95% and posterior probabilities ≥ 0.95. Tree was rooted using *Chrysochir aureus* and *Megalonibea fusca.*
